# Supplementary material for: Long seed dispersal distances by an inquisitive flightless rail (Gallirallus australis) are reduced by interaction with humans
Source: R Soc Open Sci. 2019 Aug 28;6(8):190397. doi: 10.1098/rsos.190397 (PMC6731707; doi:10.1098/rsos.190397)
Supplement: R code for mechanistic model [file rsos190397supp3.docx]

ESM 3: R code for mechanistic model

# 28/05/2018 updated 08/06/2018

# by Elena Moltchanova with edits by Jo Carpenter

rm(list=ls())

#library(geosphere)

load("~/Desktop/waypoints.Rdata")

# auxiliarly: sampling from truncated gamma

rtgamma <- function(n,a,b,upper=Inf){qgamma(runif(n)*pgamma(upper,a,b),a,b)}

# auxiliarly: haversine

hav <- function(theta){(1-cos(theta))/2}

# waypoints contain the information on individual birds: time and location

Nbirds <- length(waypoints)

# number of waypoints for each bird, maxtime (length of observation) for each bird

maxtime <- nwpts <- numeric(Nbirds)

minlat <- maxlat <- minlon <- maxlon <- numeric(Nbirds)

for(i in 1:Nbirds){

nwpts[i] <- dim(waypoints[[i]])[1]

maxtime[i] <- max(waypoints[[i]]$time)

minlat[i] <- min(waypoints[[i]]$Latitude)

maxlat[i] <- min(waypoints[[i]]$Latitude)

minlon[i] <- min(waypoints[[i]]$Longitude)

maxlon[i] <- min(waypoints[[i]]$Longitude)

}

# idea: sample a random vector of Nseeds seed retention times

# guttime measured in HOURS

set.seed(20180528)

Nseed <- 10^5

# estimating the distances travelled by the seed

dist.est <- array(dim=c(Nbirds,Nseed))

# start of bird-loop

for(bird in 1:Nbirds){print(bird)

# gut time for hinau

my.guttime.list<-c(355.51, 7.43, 7.43, 47.06, 20.46, 21, 20.46, 20.46, 103.15, 103.15, 15.16, 15.30, 15.30, 15.46, 84.45, 84.45, 2.46, 5.45, 5.45)

# I do not like truncation, let's talk censoring

guttime <- sample(my.guttime.list,size=Nseed,replace=T)

# random time of ingestion

guttime0 <- runif(Nseed,0,maxtime[bird]-guttime)

# time of exit

guttime1 <- guttime0 + guttime

# so we randomly choose guttime and randomly place it anywhere within the birds trajectory

# if it fits

guttime.ok <- (!is.na(guttime0)); n.ok <- sum(guttime.ok)

# interval during which the ingestion happens

interval0 <- apply(

(array(guttime0[guttime.ok],dim=c(n.ok,nwpts[bird]-1))>=

t(array(waypoints[[bird]]$time[-nwpts[bird]],dim=c(nwpts[bird]-1,n.ok))))&

(array(guttime0[guttime.ok],dim=c(n.ok,nwpts[bird]-1))<

t(array(waypoints[[bird]]$time[-1],dim=c(nwpts[bird]-1,n.ok)))),1,which)

### so... what are the coordinates at the beginning and end of the interval?

t00 <- waypoints[[bird]]$time[interval0]

lat00 <- waypoints[[bird]]$Latitude[interval0]

lon00 <- waypoints[[bird]]$Longitude[interval0]

t01 <- waypoints[[bird]]$time[interval0+1]

lat01 <- waypoints[[bird]]$Latitude[interval0+1]

lon01 <- waypoints[[bird]]$Longitude[interval0+1]

# so the actual coordinates are

lat0 <- lat00+(lat01-lat00)/(t01-t00)*(guttime0[guttime.ok]-t00)

lon0 <- lon00+(lon01-lon00)/(t01-t00)*(guttime0[guttime.ok]-t00)

# interval during which the exit happens

interval1 <- apply(

(array(guttime1[guttime.ok],dim=c(n.ok,nwpts[bird]-1))>=

t(array(waypoints[[bird]]$time[-nwpts[bird]],dim=c(nwpts[bird]-1,n.ok))))&

(array(guttime1[guttime.ok],dim=c(n.ok,nwpts[bird]-1))<

t(array(waypoints[[bird]]$time[-1],dim=c(nwpts[bird]-1,n.ok)))),1,which)

### so... what are the coordinates at the beginning and end of the interval?

t00 <- waypoints[[bird]]$time[interval1]

lat00 <- waypoints[[bird]]$Latitude[interval1]

lon00 <- waypoints[[bird]]$Longitude[interval1]

t01 <- waypoints[[bird]]$time[interval1+1]

lat01 <- waypoints[[bird]]$Latitude[interval1+1]

lon01 <- waypoints[[bird]]$Longitude[interval1+1]

# so the actual coordinates are

lat1 <- lat00+(lat01-lat00)/(t01-t00)*(guttime1[guttime.ok]-t00)

lon1 <- lon00+(lon01-lon00)/(t01-t00)*(guttime1[guttime.ok]-t00)

# distances (m) between two sets of coordinates

h.tmp <- hav((lat1-lat0)*pi/180)+cos(lat1*pi/180)*cos(lat0*pi/180)*hav((lon1-lon0)*pi/180)

dist.est[bird,1:n.ok] <- 2*6378137*asin(sqrt(h.tmp))

} # end of bird-loop
